# Genetically proxied glucose-lowering drug target perturbation and risk of cancer: a Mendelian randomisation analysis

**DOI:** 10.1007/s00125-023-05925-4

**Published:** 2023-05-12

**Authors:** James Yarmolinsky, Emmanouil Bouras, Andrei Constantinescu, Kimberley Burrows, Caroline J. Bull, Emma E. Vincent, Richard M. Martin, Olympia Dimopoulou, Sarah J. Lewis, Victor Moreno, Marijana Vujkovic, Kyong-Mi Chang, Benjamin F. Voight, Philip S. Tsao, Marc J. Gunter, Jochen Hampe, Andrew J. Pellatt, Paul D. P. Pharoah, Robert E. Schoen, Steven Gallinger, Mark A. Jenkins, Rish K. Pai, the PRACTICAL consortium, Rosalind A. Eeles, Christopher A. Haiman, Zsofia Kote-Jarai, Fredrick R. Schumacher, Sara Benlloch, Ali Amin Al Olama, Kenneth Muir, Sonja I. Berndt, David V. Conti, Fredrik Wiklund, Stephen Chanock, Ying Wang, Victoria L. Stevens, Catherine M. Tangen, Jyotsna Batra, Judith A. Clements, Henrik Grönberg, Nora Pashayan, Johanna Schleutker, Demetrius Albanes, Stephanie Weinstein, Alicja Wolk, Catharine M. L. West, Lorelei A. Mucci, Géraldine Cancel-Tassin, Stella Koutros, Karina Dalsgaard Sørensen, Eli Marie Grindedal, David E. Neal, Freddie C. Hamdy, Jenny L. Donovan, Ruth C. Travis, Robert J. Hamilton, Sue Ann Ingles, Barry S. Rosenstein, Yong-Jie Lu, Graham G. Giles, Adam S. Kibel, Ana Vega, Manolis Kogevinas, Kathryn L. Penney, Jong Y. Park, Janet L. Stanford, Cezary Cybulski, Børge G. Nordestgaard, Sune F. Nielsen, Hermann Brenner, Christiane Maier, Jeri Kim, Esther M. John, Manuel R. Teixeira, Susan L. Neuhausen, Kim De Ruyck, Azad Razack, Lisa F. Newcomb, Davor Lessel, Radka Kaneva, Nawaid Usmani, Frank Claessens, Paul A. Townsend, Jose Esteban Castelao, Monique J. Roobol, Florence Menegaux, Kay-Tee Khaw, Lisa Cannon-Albright, Hardev Pandha, Stephen N. Thibodeau, David J. Hunter, Peter Kraft, William J. Blot, Elio Riboli, VA Million Veteran Program, Dipender Gill, Kostas K. Tsilidis

**Affiliations:** 1 MRC Integrative Epidemiology Unit, University of Bristol, Bristol, UK; 2 Population Health Sciences, Bristol Medical School, University of Bristol, Bristol, UK; 3 Department of Hygiene and Epidemiology, University of Ioannina Medical School, Ioannina, Greece; 4 School of Translational Health Sciences, University of Bristol, Bristol, UK; 5 NIHR Bristol Biomedical Research Centre, University Hospitals Bristol and Weston NHS Foundation Trust and the University of Bristol, Bristol, UK; 6 Biomarkers and Susceptibility Unit, Oncology Data Analytics Program, Catalan Institute of Oncology (ICO), L’Hospitalet de Llobregat, Barcelona, Spain; 7 Colorectal Cancer Group, ONCOBELL Program, Bellvitge Biomedical Research Institute (IDIBELL), L’Hospitalet de Llobregat, Barcelona, Spain; 8 Consortium for Biomedical Research in Epidemiology and Public Health (CIBERESP), Madrid, Spain; 9 Department of Clinical Sciences, Faculty of Medicine, University of Barcelona, Barcelona, Spain; 10 Corporal Michael J. Crescenz VA Medical Center, Philadelphia, PA USA; 11 Department of Medicine, University of Pennsylvania Perelman School of Medicine, Philadelphia, PA USA; 12 Department of Systems Pharmacology and Translational Therapeutics, Perelman School of Medicine, University of Pennsylvania, Philadelphia, PA USA; 13 Department of Genetics, Perelman School of Medicine, University of Pennsylvania, Philadelphia, PA USA; 14 Institute of Translational Medicine and Therapeutics, University of Pennsylvania, Philadelphia, PA USA; 15 VA Palo Alto Epidemiology Research and Information Center for Genomics, VA Palo Alto Health Care System, Palo Alto, CA USA; 16 Department of Medicine, Stanford University School of Medicine, Stanford, CA USA; 17 Stanford Cardiovascular Institute, Stanford University School of Medicine, Stanford, CA USA; 18 Nutrition and Metabolism Section, International Agency for Research on Cancer, World Health Organization, Lyon, France; 19 Department of Medicine I, University Hospital Dresden, Technische Universität Dresden (TU Dresden), Dresden, Germany; 20 University of Texas MD Anderson Cancer Center, Houston, TX USA; 21 Department of Public Health and Primary Care, University of Cambridge, Cambridge, UK; 22 Department of Medicine and Epidemiology, University of Pittsburgh Medical Center, Pittsburgh, PA USA; 23 Lunenfeld Tanenbaum Research Institute, Mount Sinai Hospital, University of Toronto, Toronto, ON Canada; 24 Centre for Epidemiology and Biostatistics, Melbourne School of Population and Global Health, The University of Melbourne, Melbourne, VIC Australia; 25 Department of Laboratory Medicine and Pathology, Mayo Clinic Arizona, Scottsdale, AZ USA; 26 Department of Epidemiology and Biostatistics, School of Public Health, Imperial College London, St Mary’s Campus, London, UK

**Keywords:** ABCC8, Breast cancer, Colorectal cancer, GLP1R, Glucose-lowering drug targets, Mendelian randomisation, PPARG, Prostate cancer

## Abstract

**Aims/hypothesis:**

Epidemiological studies have generated conflicting findings on the relationship between glucose-lowering medication use and cancer risk. Naturally occurring variation in genes encoding glucose-lowering drug targets can be used to investigate the effect of their pharmacological perturbation on cancer risk.

**Methods:**

We developed genetic instruments for three glucose-lowering drug targets (peroxisome proliferator activated receptor γ [PPARG]; sulfonylurea receptor 1 [ATP binding cassette subfamily C member 8 (ABCC8)]; glucagon-like peptide 1 receptor [GLP1R]) using summary genetic association data from a genome-wide association study of type 2 diabetes in 148,726 cases and 965,732 controls in the Million Veteran Program. Genetic instruments were constructed using *cis*-acting genome-wide significant (*p*<5×10^−8^) SNPs permitted to be in weak linkage disequilibrium (*r*^2^<0.20). Summary genetic association estimates for these SNPs were obtained from genome-wide association study (GWAS) consortia for the following cancers: breast (122,977 cases, 105,974 controls); colorectal (58,221 cases, 67,694 controls); prostate (79,148 cases, 61,106 controls); and overall (i.e. site-combined) cancer (27,483 cases, 372,016 controls). Inverse-variance weighted random-effects models adjusting for linkage disequilibrium were employed to estimate causal associations between genetically proxied drug target perturbation and cancer risk. Co-localisation analysis was employed to examine robustness of findings to violations of Mendelian randomisation (MR) assumptions. A Bonferroni correction was employed as a heuristic to define associations from MR analyses as ‘strong’ and ‘weak’ evidence.

**Results:**

In MR analysis, genetically proxied PPARG perturbation was weakly associated with higher risk of prostate cancer (for PPARG perturbation equivalent to a 1 unit decrease in inverse rank normal transformed HbA_1c_: OR 1.75 [95% CI 1.07, 2.85], *p*=0.02). In histological subtype-stratified analyses, genetically proxied PPARG perturbation was weakly associated with lower risk of oestrogen receptor-positive breast cancer (OR 0.57 [95% CI 0.38, 0.85], *p*=6.45×10^−3^). In co-localisation analysis, however, there was little evidence of shared causal variants for type 2 diabetes liability and cancer endpoints in the *PPARG* locus, although these analyses were likely underpowered. There was little evidence to support associations between genetically proxied PPARG perturbation and colorectal or overall cancer risk or between genetically proxied ABCC8 or GLP1R perturbation with risk across cancer endpoints.

**Conclusions/interpretation:**

Our drug target MR analyses did not find consistent evidence to support an association of genetically proxied PPARG, ABCC8 or GLP1R perturbation with breast, colorectal, prostate or overall cancer risk. Further evaluation of these drug targets using alternative molecular epidemiological approaches may help to further corroborate the findings presented in this analysis.

**Data availability:**

Summary genetic association data for select cancer endpoints were obtained from the public domain: breast cancer (https://bcac.ccge.medschl.cam.ac.uk/bcacdata/); and overall prostate cancer (http://practical.icr.ac.uk/blog/). Summary genetic association data for colorectal cancer can be accessed by contacting GECCO (kafdem at fredhutch.org). Summary genetic association data on advanced prostate cancer can be accessed by contacting PRACTICAL (practical at icr.ac.uk). Summary genetic association data on type 2 diabetes from Vujkovic et al (Nat Genet, 2020) can be accessed through dbGAP under accession number phs001672.v3.p1 (pha004945.1 refers to the European-specific summary statistics). UK Biobank data can be accessed by registering with UK Biobank and completing the registration form in the Access Management System (AMS) (https://www.ukbiobank.ac.uk/enable-your-research/apply-for-access).

**Graphical Abstract:**

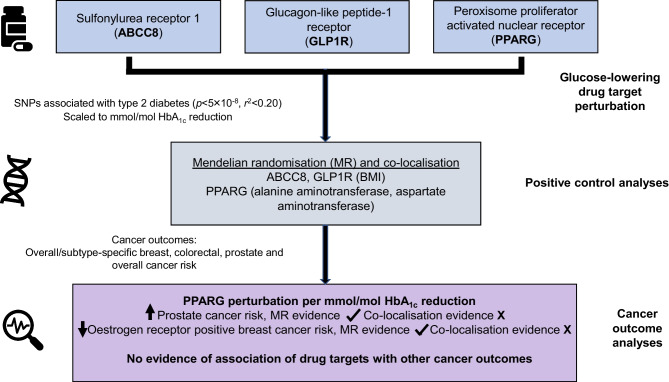

**Supplementary Information:**

The online version contains peer-reviewed but unedited supplementary material available at 10.1007/s00125-023-05925-4.


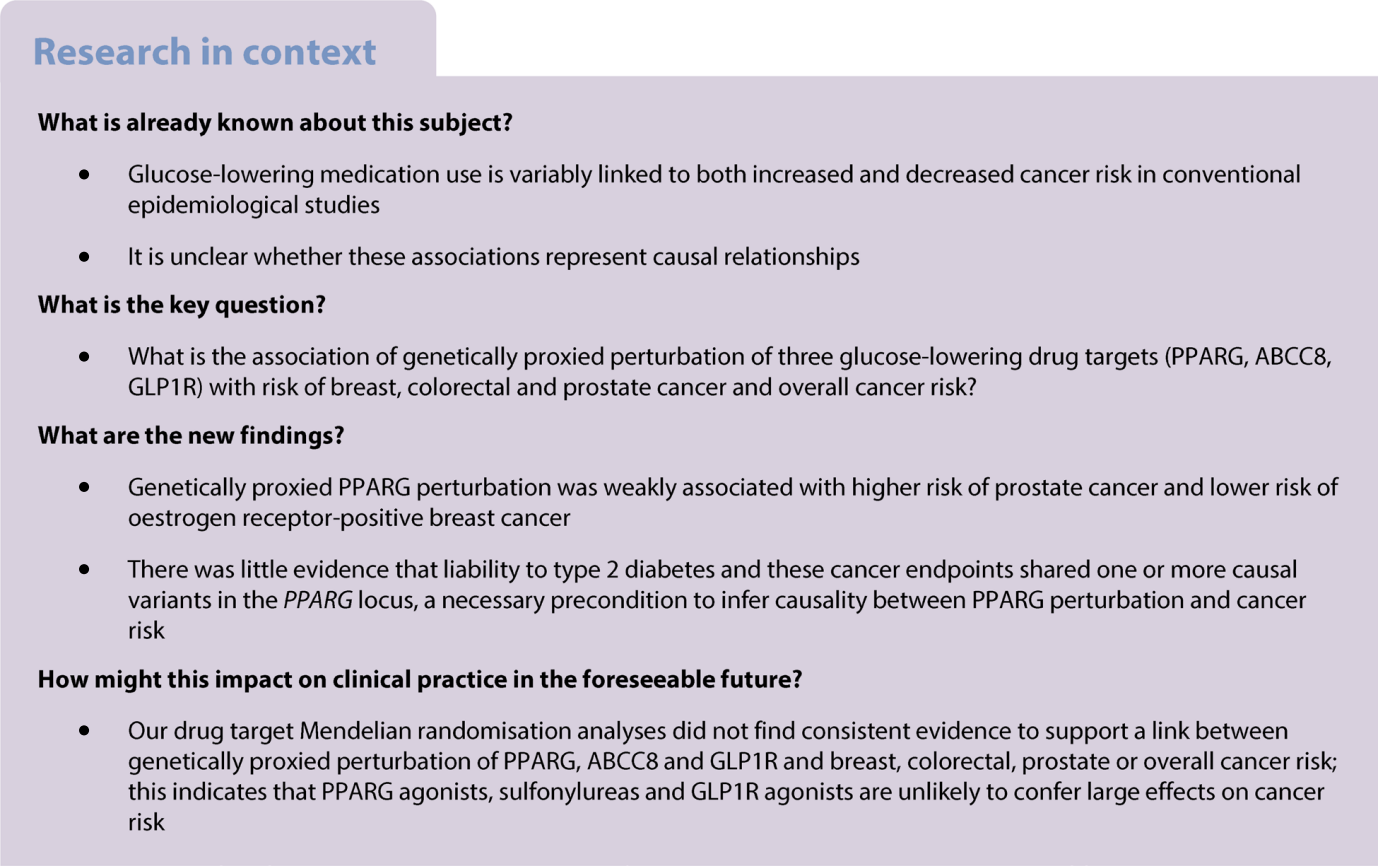


## Introduction

Globally, an estimated 460 million individuals have type 2 diabetes, most of whom require long-term use of glucose-lowering medications to maintain glycaemic control [1]. Several different classes of oral glucose-lowering medications are used to manage this condition, including biguanides (e.g. metformin), sulfonylureas, thiazolidinediones, dipeptidyl peptidase-4 (DPP-4) inhibitors, sodium–glucose cotransporter 2 (SGLT2) inhibitors and glucagon-like peptide-1 receptor (GLP1R) agonists, with diverse mechanisms of action [2].

Preclinical studies have variably reported both carcinogenic and antineoplastic effects of glucose-lowering medications. For example, in vitro studies have suggested that metformin, an insulin sensitiser and first-line therapy for type 2 diabetes, can reduce cell proliferation, induce apoptosis and cause cell cycle arrest [3]. Thiazolidinediones, insulin sensitisers and selective peroxisome proliferator activated nuclear receptor (PPARG) agonists have been suggested to increase cellular differentiation, reduce cellular proliferation and induce apoptosis in some cell lines but to promote metastatic prostate cancer in vivo [4–6]. There is also some evidence that sulfonylureas, secretagogues that lower blood glucose levels by stimulating pancreatic insulin secretion, may promote carcinogenesis, potentially via increasing circulating insulin levels [7, 8]. Finally, in vitro studies have reported potential antiproliferative effects of GLP1R agonists in various cancer cell types [9–11].

Epidemiological studies of glucose-lowering medication use have provided some support for findings from laboratory studies. For example, some observational studies have reported that metformin users have lower risk of several cancers while sulfonylurea use has been associated with an increased risk of site-specific (i.e. colorectal, metastatic prostate) and overall cancer [12–17]. In addition, some thiazolidinediones (i.e. pioglitazones) have been linked to an elevated risk of bladder, prostate and pancreatic cancer, though use of rosiglitazone has been associated with lower breast cancer risk [18, 19]. Finally, GLP1R agonist use has been associated with a decreased risk of prostate cancer when compared with sulfonylurea use [20].

The causal nature of associations reported between glucose-lowering medication use and cancer risk in conventional epidemiological studies is often unclear. This is because of the susceptibility of such studies to residual confounding (e.g. due to indication) and various forms of bias (e.g. immortal time, prevalent user), which can undermine robust causal inference [21]. While clinical trials of glucose-lowering medications have not consistently reported differences in rates of cancer among users of these medications, such studies are often underpowered to detect effects for individual cancer sites [22, 23]. Further, such studies often have limited follow-up periods, thus are not able to adequately capture outcomes with long induction periods, such as cancer.

Drug target Mendelian randomisation (MR) uses germline variants in genes encoding drug targets as instruments (‘proxies’) for these targets to estimate the effect of their pharmacological perturbation on disease endpoints [24]. Since germline genetic variants are randomly assorted at meiosis and fixed at conception, analyses using variants as instruments should be less prone to conventional issues of confounding and reverse causation. In addition, given the length of time required for solid tumour development, the use of germline genetic variants as instruments is advantageous as it permits estimation of the long-term effects of medication use on cancer risk [25].

Given the widespread use of glucose-lowering medications and reports of both adverse and protective associations of these medications with cancer risk in preclinical and epidemiological studies, there is a need to further evaluate the role of these medications in the risk of common adulthood cancers. Additionally, given the long induction period of cancers, using MR to examine target-mediated effects of medications that have been on the market for relatively short periods of time (e.g. SGLT2 inhibitors and GLP1R agonists) can be informative in predicting their long-term safety profiles. We thus aimed to develop genetic instruments for the targets of five approved type 2 diabetes medications with known mechanisms of action (sulfonylurea receptor 1 [ATP binding cassette subfamily C member 8 (ABCC8)], PPARG, SGLT2, DPP4 and GLP1R). We also aimed to evaluate associations of genetically proxied perturbation of three of these targets with reliable *cis*-acting instruments (ABCC8, PPARG and GLP1R) with risk of breast, colorectal and prostate cancer, common cancers with epidemiological evidence suggesting a link between glucose-lowering medication use and their onset, and overall (i.e. site-combined) cancer [5, 12–14, 18, 19, 26–28].

## Methods

Summary genetic association data were obtained from three cancer-specific genome-wide association study (GWAS) consortia. Summary genetic association estimates for overall and oestrogen receptor (ER)-stratified breast cancer risk in up to 122,977 cases and 105,974 controls were obtained from the Breast Cancer Association Consortium (BCAC) [29]. Summary genetic association estimates for overall and site-specific (i.e. colon, rectal) colorectal cancer risk in up to 58,221 cases and 67,694 controls were obtained from an analysis of the Genetics and Epidemiology of Colorectal Cancer Consortium (GECCO), Colorectal Transdisciplinary Study (CORECT), and Colon Cancer Family Registry (CCFR) [30]. Summary genetic association estimates for overall and advanced prostate cancer risk (i.e. metastatic disease, Gleason score ≥8, prostate-specific antigen >100 or prostate cancer-related death) in up to 79,148 cases and 61,106 controls were obtained from the Prostate Cancer Association Group to Investigate Cancer Associated Alterations in the Genome (PRACTICAL) consortium [31]. These analyses were restricted to participants of European ancestry.

Overall (i.e. site-combined) cancer risk data in 27,483 incident cases and 372,016 controls were also obtained from a GWAS performed in the UK Biobank cohort study [32]. Briefly, cancer cases were classified according to ICD-9 (http://www.icd9data.com/2007/Volume1/default.htm) and ICD-10 (http://apps.who.int/classifications/icd10/browse/2016/en) with data completed to April 2019 and controls were defined as individuals who did not have any cancer code (ICD9 or ICD10) and did not self-report a cancer diagnosis. GWAS were performed using a linear mixed model as implemented in BOLT-LMM (v2.3) (to account for relatedness and population stratification) and adjusted for age, sex and genotyping array [33]. Further information on imputation and quality control measures have been reported elsewhere [33].

Further information on statistical analysis, imputation, and quality control measures for summary genetic association data obtained from cancer consortia is available in the original publications. All studies contributing data to these analyses had the relevant institutional review board approval from each country, in accordance with the Declaration of Helsinki, and all participants provided informed consent.

### Instrument construction

To generate genetic instruments to proxy glucose-lowering drug target perturbation, summary genetic association data were obtained from a GWAS of type 2 diabetes in the Million Veteran Program (148,726 cases; 965,732 controls of European ancestry) [34]. Analyses were adjusted for age, sex and ten principal components of genetic ancestry. Instruments were constructed in PLINK by obtaining SNPs associated with type 2 diabetes at genome-wide significance (*p*<5×10^−8^) that were in or within ±500 kb from the gene encoding each respective target (PPARG, Chr3: 12328867–12475855; ABCC8, Chr11: 17414432–17498449; GLP1R, Chr6: 39016574–39055519) using the 1000 Genomes Phase 3 reference panel [35, 36]. We were unable to identify genome-wide significant SNPs within 500 kb windows from *SLC5A2* and *DPP4* (i.e. instruments for SGLT2 and DPP-4 inhibitors, respectively) and therefore did not proceed with MR analyses for these targets. We also did not include putative metformin targets due to the unclear mechanism(s) of action of this medication [37]. For PPARG, ABCC8 and GLP1R, SNPs used as instruments were permitted to be in weak linkage disequilibrium (*r*^2^<0.20) with each other to increase the proportion of variance in each respective drug target explained by the instrument, maximising instrument strength. In total, nine SNPs that met these criteria were obtained for PPARG, six for ABCC8 and four for GLP1R.

In a separate population (i.e. the UK Biobank cohort study), we then evaluated the association of type 2 diabetes SNPs in drug target regions with HbA_1c_ levels, a marker of long-term blood glucose levels, in order to minimise winner’s curse bias. The UK Biobank is a prospective cohort study of ~500,000 individuals aged 40–69 years when recruited in 2006–2010 [38]. SNP summary statistics were re-scaled to represent a mmol/mol (0.09%) unit reduction in HbA_1c_ to provide more interpretable effect estimates in MR analyses. HbA_1c_ values were obtained from a GWAS of 407,766 participants of the UK Biobank performed using a linear mixed model as implemented in BOLT-LMM and adjusted for age, sex, batch and ten principal components of genetic ancestry. For the purposes of this analysis, we sequentially removed participants according to the following exclusion criteria: withdrawn from the study (*N*=502,506 retained); non-European ancestry (*N*=462,898 retained); missing HbA_1c_ data (*N*=442,529 retained); missing or ‘prefer not to answer’ response to self-reported diabetes status (*N*=442,268); self-reported diabetes diagnosis (*N*=418,574); ICD-10 diabetes diagnosis (*N*=409,812); missing data on glucose-lowering medication use (*N*=409,762); self-reported glucose-lowering medication use (*N*=409,614); HbA_1c_ >48 mmol/mol (6.5%) (*N*=408,319); and HbA_1c_ <21.88 mmol/mol (4.2%) (*N*=407,766). Further information on imputation and quality control measures have been reported elsewhere [39].

For the PPARG instrument, two SNPs where the effect on HbA_1c_ was in the opposite direction to that of type 2 diabetes were removed from the instrument (rs17036160, rs11712085), as these associations likely represent pleiotropic mechanisms that would bias consequent MR analyses.

### Instrument validation

Instruments were validated by examining the association of genetically proxied drug target perturbation with endpoints influenced by these medications in randomised controlled trials. For PPARG, alanine aminotransferase (ALT) and aspartate aminotransferase (AST) levels were used as positive controls (i.e. PPARG agonists lower levels of ALT and AST) and for ABCC8 and GLP1R, BMI was used (i.e. sulfonylureas cause weight gain and GLP1R agonists cause weight loss) [40–43]. Co-localisation was then performed to assess whether genetic liability to type 2 diabetes and traits representing positive controls share the same causal variant at each locus encoding a drug target (i.e. *PPARG*, *ABCC8*, *GLP1R*). Such an analysis can permit exploration of whether genetic liability to type 2 diabetes and positive control traits at each drug target locus are influenced by distinct causal variants that are in linkage disequilibrium with each other, indicative of horizontal pleiotropy (an instrument influencing an outcome through pathways independent to that of the exposure), a violation of the exclusion restriction criterion.

Co-localisation analysis was performed using the coloc (version 2.0) R package (https://cran.r-project.org/web/packages/coloc/index.html), which uses approximate Bayes factor computation to generate posterior probabilities that associations between two traits represent each of the following configurations: (1) neither trait has a genetic association in the region (H_0_); (2) only the first trait has a genetic association in the region (H_1_); (3) only the second trait has a genetic association in the region (H_2_); (4) both traits are associated but have different causal variants (H_3_); and (5) both traits are associated and share a single causal variant (H_4_) [44]. Co-localisation analysis was performed by generating ±500 kb windows around the gene encoding each respective drug target. We used a posterior probability of >50% to indicate support for a configuration tested. Where there was not support for H_4_, we then examined the possibility of co-localisation across other secondary conditionally independent signals for either genetic liability to type 2 diabetes or positive controls within drug target loci by performing pairwise conditional and co-localisation analysis on all conditionally independent association signals using GCTA-COJO and the coloc package as implemented in pwCoCo [45]. We employed default priors for p1 (i.e. prior probability that a SNP is associated with type 2 diabetes liability within a drug target locus, 1×10^−4^), p2 (i.e. prior probability that a SNP is associated with positive controls or cancer risk within a drug target locus, 1×10^−4^) and p12 (i.e. prior probability that a SNP is associated with both traits, 1×10^−5^). As sensitivity analyses, we re-performed co-localisation analysis employing two alternate priors for p12 (5×10^−5^, 5×10^−6^).

### Statistical analysis

Causal estimates were generated using inverse-variance weighted (IVW) random-effects models (permitting overdispersion in models). These models were adjusted for weak linkage disequilibrium between SNPs with reference to the 1000 Genomes Phase 3 reference panel [46]. Where there was under-dispersion in causal estimates generated from individual genetic variants, the residual SE was set to 1 (i.e. equivalent to a fixed-effects model).

MR analysis makes the following assumptions: (1) that a genetic instrument is associated with a modifiable exposure or drug target (‘relevance’); (2) the instrument does not share a common cause with an outcome (‘exchangeability’); and (3) the instrument has no direct effect on the outcome (‘exclusion restriction’).

The ‘relevance’ MR assumption was evaluated by generating estimates of the proportion of variance of each drug target (in HbA_1c_ units) explained by the instrument (*r*^2^) and *F* statistics. *F* statistics can be used to examine whether results are likely to be influenced by weak instrument bias (i.e. reduced statistical power and bias when an instrument explains a limited proportion of the variance in a drug target). As a convention, an *F* statistic of >10 is indicative of minimal weak instrument bias.

We evaluated the ‘exclusion restriction’ MR assumption by performing co-localisation to examine whether drug targets and cancer endpoints showing nominal evidence of an association in MR analyses (*p*<0.05) share the same causal variant at a given locus. Iterative leave-one-out analysis was performed by removing one SNP at a time from instruments to examine whether findings showing nominal evidence of association were driven by a single influential SNP.

To account for multiple testing across analyses, a Bonferroni correction was used to establish a *p* value threshold of <0.0019 (false-positive rate = 0.05/27 statistical tests [three drug targets tested against nine primary cancer endpoints]), which we used as a heuristic to define ‘strong evidence’, with findings between *p*≥0.0019 and *p*<0.05 defined as ‘weak evidence’.

There was no formal prespecified protocol for this study. All statistical analyses were performed using R version 3.3.1 (https://www.r-project.org/).

## Results

Characteristics of genetic variants used to instrument glucose-lowering drug targets are presented in Table 1. Across all three drug targets, *F* statistics for their respective instruments ranged from 56.32 to 487.14, suggesting that weak instrument bias was unlikely to affect the conclusions (ESM Table 1). Power calculations suggested that we had 80% power to detect ORs ranging from 1.40 to 2.62 (in PPARG analyses), 2.03 to 8.34 (in ABCC8 analyses) and 2.22 to 8.78 (in GLP1R analyses) per mmol/mol reduction in target-mediated inverse rank normal transformed [IRNT] HbA_1c_ across all cancer endpoints (α=0.05). Complete power estimates across all MR analyses are presented in ESM Table 2.

**Table 1:** Characteristics of SNPs used as instruments to proxy drug targets

SNP	EA/NEA	EAF	Effect (SE) T2D	*p* value T2D	Effect (SE) HbA_1c_
ABCC8					
rs5219	C/T	0.63	–0.069 (0.005)	3.15×10^−48^	–0.016 (0.002)
rs4148640	T/G	0.27	–0.045 (0.007)	4.47×10^−11^	–0.009 (0.002)
rs61880293	T/C	0.92	–0.058 (0.009)	8.13×10^−10^	–0.015 (0.004)
rs7130826	T/G	0.73	–0.031 (0.005)	1.94×10^−9^	–0.004 (0.002)
rs10832783	G/A	0.92	–0.055 (0.009)	4.63×10^−9^	–0.011 (0.004)
rs214080	A/G	0.41	–0.026 (0.005)	1.96×10^−8^	–0.007 (0.002)
GLP1R					
rs10305420	T/C	0.38	–0.032 (0.005)	2.69×10^−11^	–0.011 (0.002)
rs34179517	A/C	0.87	–0.044 (0.007)	3.09×10^−10^	–0.007 (0.003)
rs9296291	C/T	0.23	–0.033 (0.006)	3.42×10^−9^	–0.013 (0.002)
rs10305457	C/T	0.90	–0.044 (0.008)	3.04×10^−8^	–0.018 (0.003)
PPARG					
rs7637403	A/G	0.11	–0.074 (0.007)	1.03×10^−24^	–0.007 (0.003)
rs4135247	A/G	0.57	–0.042 (0.005)	2.23×10^−19^	–0.035 (0.002)
rs598747	A/G	0.84	–0.052 (0.007)	9.51×10^−15^	–0.022 (0.003)
rs150535373	A/G	0.02	–0.140 (0.019)	5.38×10^−13^	–0.005 (0.009)
rs143888770	T/C	0.02	–0.106 (0.016)	1.21×10^−10^	–0.015 (0.007)
rs17819328	T/G	0.58	–0.028 (0.005)	9.56×10^−10^	–0.023 (0.002)
rs4135300	C/T	0.89	–0.045 (0.008)	5.50×10^−9^	–0.013 (0.003)

Effect (SE) corresponds to change in log_*e*_ (OR) of type 2 diabetes or change in IRNT HbA_1c_ (mmol/mol)

*p* value corresponds to type 2 diabetes analyses

EA, effect allele; EAF, effect allele frequency; NEA, non-effect allele; T2D, type 2 diabetes

### Instrument validation

**Fig. 1:**
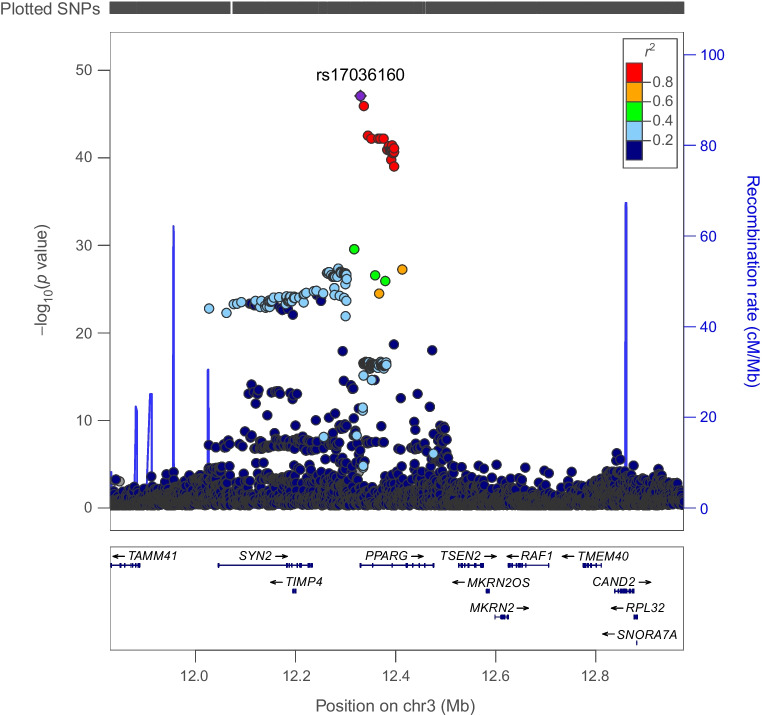
Regional Manhattan plot of associations of SNPs with type 2 diabetes ±500 kb from the *PPARG* locus. rs17036160 (purple dot) represents the sentinel SNP associated with genetic liability to type 2 diabetes in the *PPARG* locus

**Fig. 2:**
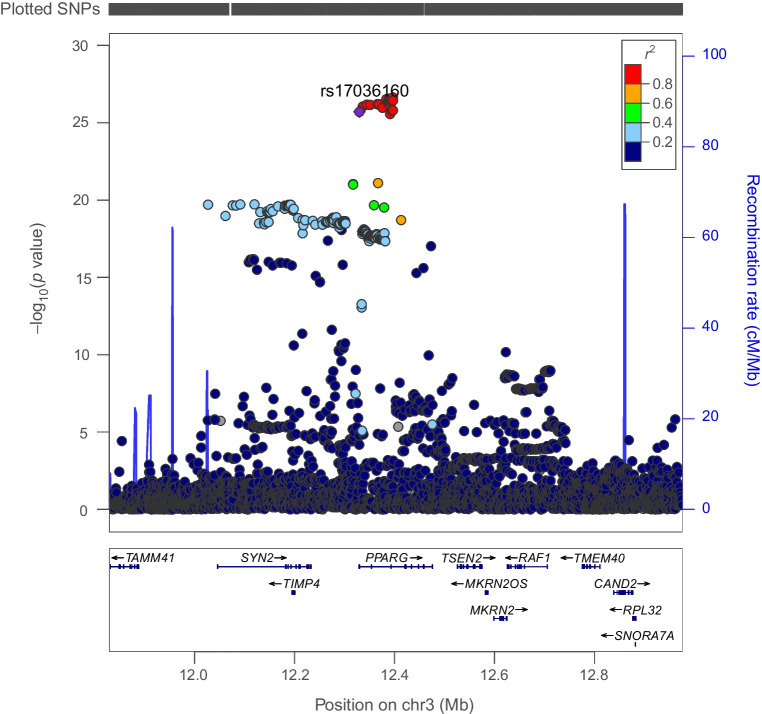
Regional Manhattan plot of associations of SNPs with ALT concentrations ±500 kb from the *PPARG* locus. rs17036160 (purple dot) represents the sentinel SNP associated with genetic liability to type 2 diabetes in the *PPARG* locus. SNPs in unclear linkage disequilibrium with sentinel SNP are in grey

**Fig. 3:**
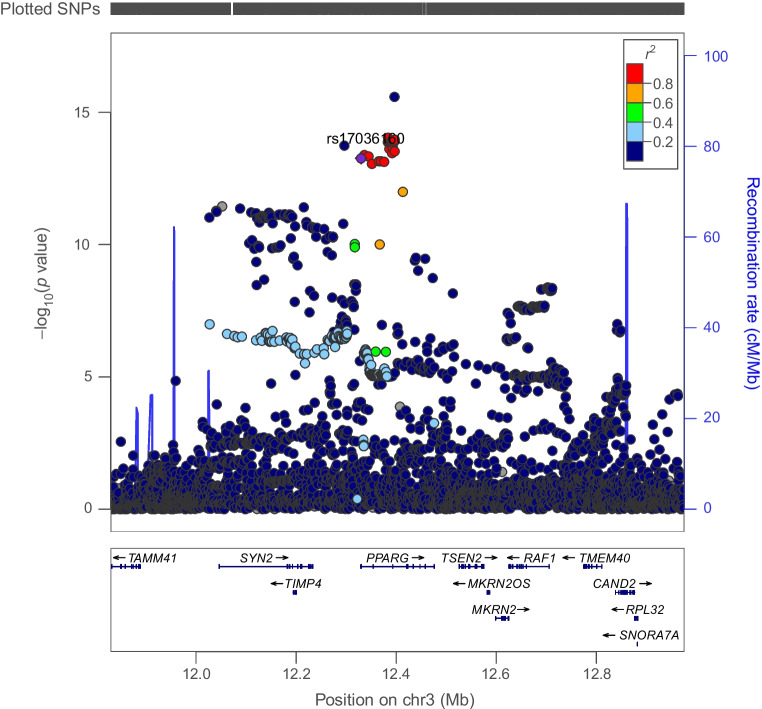
Regional Manhattan plot of associations of SNPs with AST concentrations ±500 kb from the *PPARG* locus. rs17036160 (purple dot) represents the sentinel SNP associated with genetic liability to type 2 diabetes in the *PPARG* locus

Genetically proxied PPARG perturbation was associated with lower levels of ALT (SD change in ALT per PPARG perturbation equivalent to 1 unit IRNT HbA_1c_ reduction: −0.57 [95% CI −1.01, −0.13], *p*=0.01) and AST (−0.49 [95% CI −1.79, −0.19], *p*=1.53×10^−3^). Co-localisation analysis suggested that type 2 diabetes associations in the *PPARG* locus had a 92% and 84% probability of sharing a causal variant with ALT and AST, respectively (Figs 1, 2 and 3 and ESM Tables 3, 4).

**Fig. 4:**
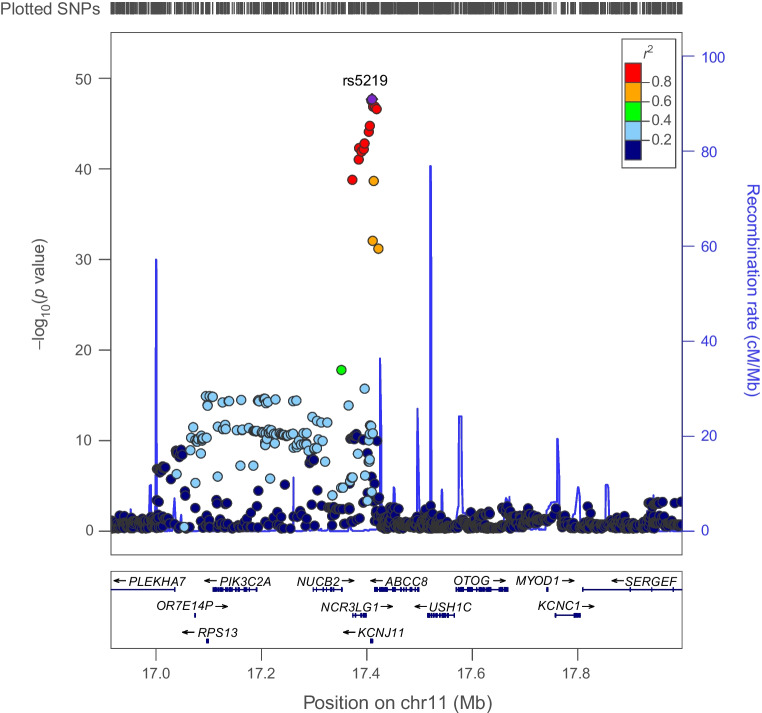
Regional Manhattan plot of associations of SNPs with type 2 diabetes ±500 kb from the *ABCC8* locus. rs5219 (purple dot) represents the sentinel SNP associated with genetic liability to type 2 diabetes in the *ABCC8* locus

**Fig. 5:**
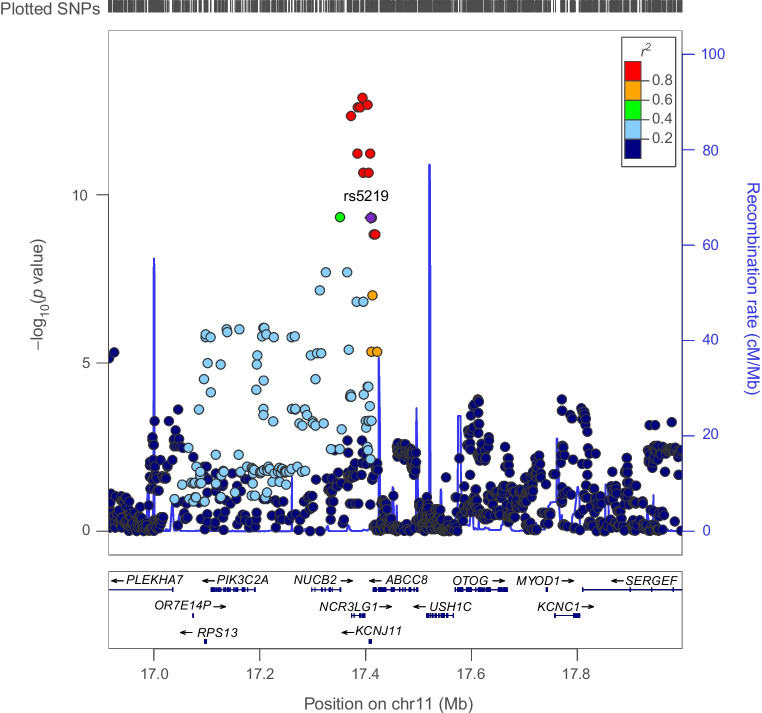
Regional Manhattan plot of associations of SNPs with BMI ±500 kb from the *ABCC8* locus. rs5219 (purple dot) represents the sentinel SNP associated with genetic liability to type 2 diabetes in the *ABCC8* locus

Genetically proxied ABCC8 perturbation was associated with elevated BMI (SD change in BMI per ABCC8 perturbation equivalent to 1 unit IRNT HbA_1c_ reduction: 0.530 [95% CI 0.004, 0.172], *p*=3.75×10^−3^). Co-localisation analysis suggested that type 2 diabetes and BMI associations had a 94.0% posterior probability of sharing a causal variant in *ABCC8* (Figs 4, 5 and ESM Table 5).

**Fig. 6:**
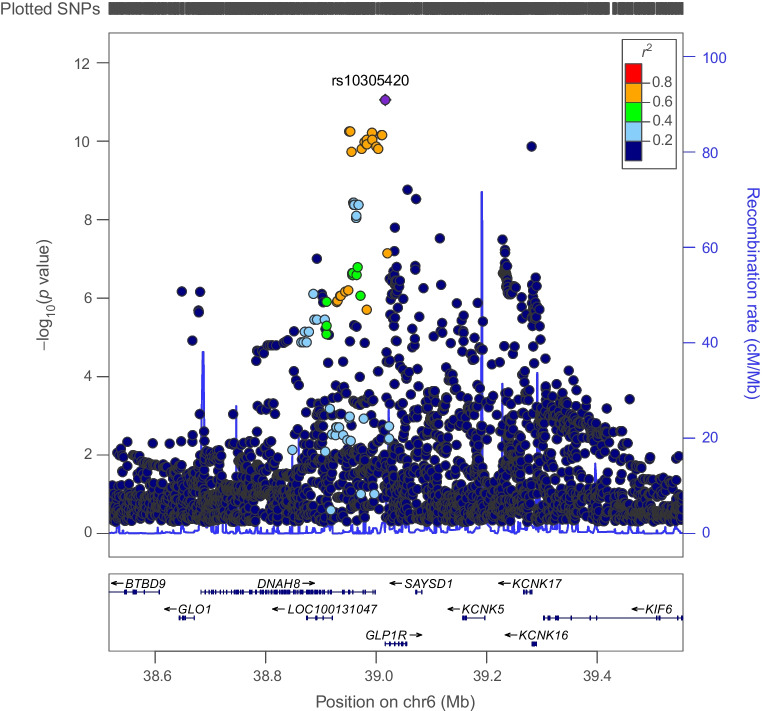
Regional Manhattan plot of associations of SNPs with type 2 diabetes ±500 kb from the *GLP1R* locus. rs10305420 (purple dot) represents the sentinel SNP associated with genetic liability to type 2 diabetes in the *GLP1R* locus

**Fig. 7:**
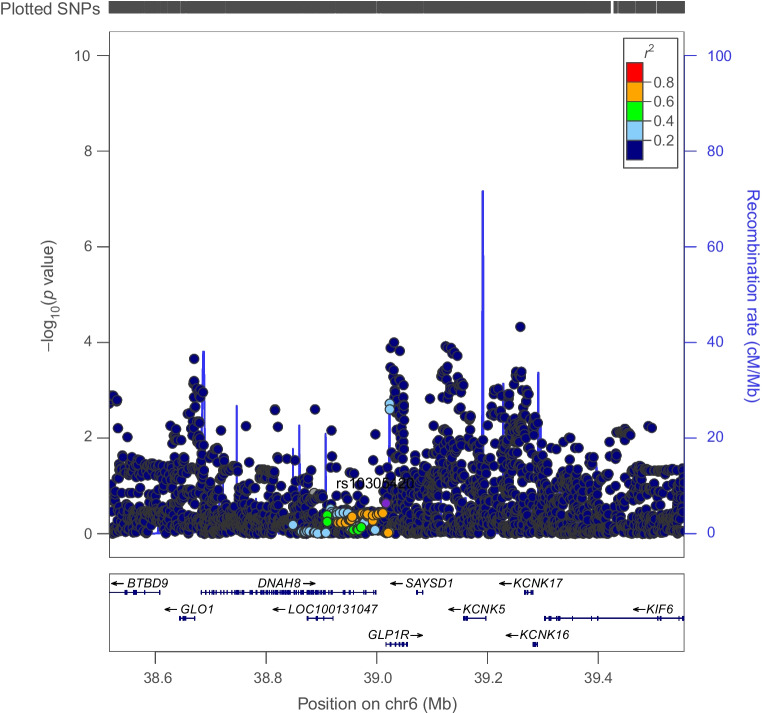
Regional Manhattan plot of associations of SNPs with BMI ±500 kb from the *GLP1R* locus. rs10305420 (purple dot) represents the sentinel SNP associated with genetic liability to type 2 diabetes in the *GLP1R* locus

There was little evidence to support an association of genetically proxied GLP1R perturbation with BMI (SD change in BMI equivalent to 1 unit IRNT HbA_1c_ reduction: −0.08 [95% CI −0.30, 0.15], *p*=0.51). Co-localisation analysis applied to both marginal and conditionally independent associations for type 2 diabetes and BMI in the *GLP1R* locus did not support shared causal variants across these traits (posterior probability of shared causal variants across models: 0.22–0.49%) (Figs 6, 7 and ESM Table 6).

### Genetically proxied PPARG perturbation and cancer risk

**Fig. 8:**
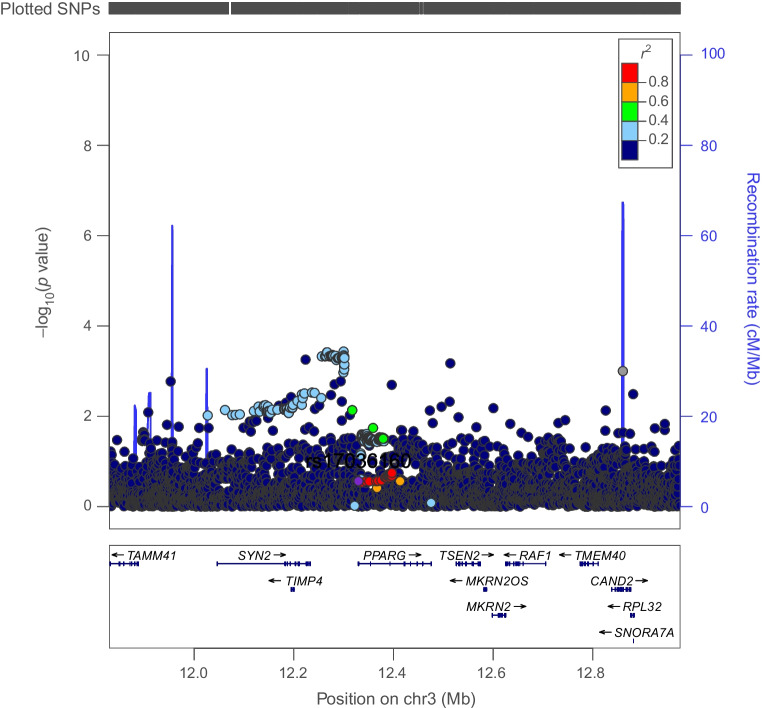
Regional Manhattan plot of associations of SNPs with prostate cancer risk ±500 kb from the *PPARG* locus. rs17036160 (purple dot) represents the sentinel SNP associated with genetic liability to type 2 diabetes in the *PPARG* locus

There was weak evidence for an association of genetically proxied PPARG perturbation with an elevated risk of prostate cancer (OR 1.75 [95% CI 1.07, 2.85], *p*=0.02) but little evidence of association with other cancer endpoints (Table 2). Findings for prostate cancer risk were consistent in iterative leave-one-out analysis (ESM Table 7). Co-localisation using marginal and conditional associations for type 2 diabetes and prostate cancer in the *PPARG* locus suggested that type 2 diabetes was unlikely to share a causal variant with this cancer in this region (posterior probability of a shared causal variant across models: ≤0.09%, posterior probability of distinct causal variants: ≤25%) (Fig. 8 and ESM Table 8).

**Table 2:** MR estimates examining the association of genetically proxied perturbation of PPARG with site-specific and overall cancer risk

Outcome	*N* (cases; controls)	OR (95% CI)	*p* value
Breast cancer	122,977; 105,974	0.67 (0.43, 1.04)	0.08
ER^+^ breast cancer	69,501; 105,974	0.57 (0.38, 0.85)	6.45×10^−3^
ER^−^ breast cancer	21,468; 105,974	1.14 (0.64, 2.01)	0.66
Colorectal cancer	58,221; 67,694	0.95 (0.51, 1.75)	0.86
Colon cancer	32,002; 64,159	1.22 (0.72, 2.08)	0.46
Rectal cancer	16,212; 64,159	0.82 (0.25, 2.71)	0.75
Prostate cancer	79,148; 61,106	1.75 (1.07, 2.85)	0.02
Advanced prostate cancer^a^	15,167; 58,308	1.64 (0.62, 4.33)	0.32
Overall cancer risk	27,483; 372,016	0.72 (0.44, 1.19)	0.20

ORs (95% CIs) are scaled to represent the effect of genetically proxied perturbation of PPARG equivalent to a 1 unit lowering of IRNT HbA_1c_ (mmol/mol)

^a^Advanced prostate cancer is defined as metastatic disease, Gleason score ≥8, prostate-specific antigen >100 or prostate cancer-related death

**Fig. 9:**
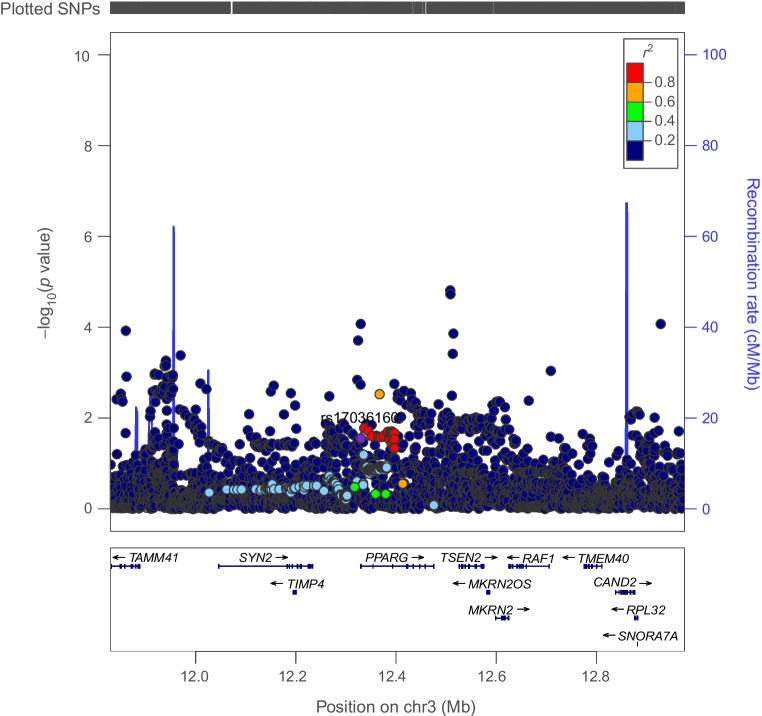
Regional Manhattan plot of associations of SNPs with ER^+^ breast cancer risk ±500 kb from the *PPARG* locus. rs17036160 (purple dot) represents the sentinel SNP associated with genetic liability to type 2 diabetes in the *PPARG* locus

In subtype-stratified analyses, genetically proxied PPARG perturbation was weakly associated with lower risk of ER^+^ breast cancer (OR 0.57 [95% CI 0.38, 0.85], *p*=6.45×10^−3^). This finding was consistent in iterative leave-one-out analysis (ESM Table 9). Co-localisation using marginal and conditional associations for type 2 diabetes and ER^+^ breast cancer in the *PPARG* locus reported a low posterior probability (H_4_<5%; posterior probability of distinct causal variants: ≤23%) of both traits sharing one or more causal variants within this region (Fig. 9 and ESM Table 10).

### Genetically proxied ABCC8 and GLP1R perturbation and cancer risk

There was little MR evidence of association of genetically proxied ABCC8 or GLP1R perturbation with site-specific or overall cancer risk (Tables 3, 4).

**Table 3:** MR estimates examining the association of genetically proxied perturbation of ABCC8 with site-specific and overall cancer risk

Outcome	*N* (cases; controls)	OR (95% CI)	*p* value
Breast cancer	122,977; 105,974	2.09 (0.81, 5.39)	0.13
ER^+^ breast cancer	69,501; 105,974	2.10 (0.66, 6.74)	0.21
ER^−^ breast cancer	21,468; 105,974	1.83 (0.48, 6.97)	0.38
Colorectal cancer	58,221; 67,694	0.61 (0.21, 1.76)	0.36
Colon cancer	32,002; 64,159	0.55 (0.15, 1.93)	0.35
Rectal cancer	16,212; 64,159	0.76 (0.15, 3.87)	0.74
Prostate cancer	79,148; 61,106	0.94 (0.37, 2.43)	0.91
Advanced prostate cancer^a^	15,167; 58,308	1.67 (0.32, 8.57)	0.54
Overall cancer risk	27,483; 372,016	0.90 (0.31, 2.59)	0.85

ORs (95% CIs) are scaled to represent the effect of genetically proxied perturbation of ABCC8 equivalent to a 1 unit lowering of IRNT HbA_1c_ (mmol/mol)

^a^Advanced prostate cancer is defined as metastatic disease, Gleason score ≥8, prostate-specific antigen >100 or prostate cancer-related death

**Table 4:** MR estimates examining the association of genetically proxied perturbation of GLP1R with site-specific and overall cancer risk

Outcome	*N* (cases; controls)	OR (95% CI)	*p* value
Breast cancer	122,977; 105,974	0.72 (0.33, 1.58)	0.42
ER^+^ breast cancer	69,501; 105,974	0.81 (0.33, 2.01)	0.65
ER^−^ breast cancer	21,468; 105,974	0.48 (0.13, 1.71)	0.26
Colorectal cancer	58,221; 67,694	1.36 (0.50, 3.68)	0.55
Colon cancer	32,002; 64,159	1.94 (0.59, 6.33)	0.27
Rectal cancer	16,212; 64,159	1.13 (0.25, 5.23)	0.87
Prostate cancer	79,148; 61,106	0.87 (0.35, 2.14)	0.76
Advanced prostate cancer^a^	15,167; 58,308	0.99 (0.10, 9.51)	0.99
Overall cancer risk	27,483; 372,016	1.21 (0.45, 3.26)	0.70

ORs (95% CIs) are scaled to represent the effect of genetically proxied perturbation of GLP1R equivalent to a 1 unit lowering of IRNT HbA_1c_ (mmol/mol)

^a^Advanced prostate cancer is defined as metastatic disease, Gleason score ≥8, prostate-specific antigen >100 or prostate cancer-related death

### Sensitivity analyses altering priors for co-localisation

Across positive control traits and cancer outcomes, findings from co-localisation analyses remained robust to using two alternate priors for p12 (5×10^−5^, 5×10^−6^) (ESM Table 11).

## Discussion

In this MR analysis of up to 287,829 cases and 606,790 controls, we found weak evidence for an association of genetically proxied PPARG perturbation with a higher risk of prostate cancer and lower risk of ER^+^ breast cancer. In co-localisation analysis, however, there was little evidence that genetic liability to type 2 diabetes and these cancer endpoints shared one or more causal variants within *PPARG*, though these analyses were likely underpowered given low posterior probabilities to support both H_3_ (i.e. distinct causal variants) and H_4_ (i.e. shared causal variants) across these analyses. We found little evidence of association of genetically proxied GLP1R or ABCC8 perturbation with cancer risk.

Despite in vivo studies suggesting an important role for PPARG in prostate tumour growth and conventional epidemiological studies suggesting a link between pioglitazone use and elevated prostate cancer risk, our combined MR and co-localisation analyses did not find consistent evidence for an association of genetically proxied PPARG perturbation with prostate cancer risk [6, 18]. Likewise, our findings are not consistent with some previous epidemiological studies that have reported links between rosiglitazone use and lower breast cancer risk and thiazolidinedione use and lower colorectal cancer risk [5, 19]. Though our analyses were powered to detect effect sizes comparable with those reported in some previous studies (e.g. ~60% increased prostate cancer risk among pioglitazone users and ~60% lower risk of colorectal cancer among thiazolidinedione users), they were likely less powered to detect other, more modest, effect sizes reported in the literature (e.g. ~10% lower risk of breast cancer in rosiglitazone users) [19, 26, 47]. Interpretation of the pharmacoepidemiological literature linking glucose-lowering medication use with cancer risk is challenging because of the likely susceptibility of many previous studies to residual confounding (e.g. by indication) due to the use of inappropriate comparator groups (i.e. non-medication users), the inclusion of ‘prevalent users’ of medications in analyses and the possibility of ‘immortal time’ bias arising due to misalignment of the start of follow-up, eligibility and treatment assignment of participants [21].

Among the strengths of our analysis is the strict instrument selection and validation process employed. By using *cis*-acting variants, in close proximity to the genes that code for the drug targets of interest, horizontal pleiotropy should be minimised. In addition, we used strict positive control analysis (i.e. testing drug targets against established secondary effects of medications) and co-localisation analyses (including co-localisation analyses permitting multiple causal variants) to validate the selected instruments. Our use of a summary-data MR approach permitted us to leverage large-scale genetic data from several GWAS consortia, enhancing statistical power and precision of causal estimates.

There were several limitations to this analysis. First, we had sufficient statistical power to detect large effect sizes only per SD decrease in HbA_1c_ (~6.75 mmol/mol [~0.61%]) and therefore cannot rule out more modest effects of the drug targets examined on cancer risk. In clinical trials, monotherapy with sulfonylureas, thiazolidinediones (rosiglitazone, pioglitazone) and the GLP1R agonist liraglutide has been shown to reduce HbA_1c_ by around 8–17 mmol/mol (0.7–1.5%), as compared with placebo [48–50]. Second, although co-localisation analyses of PPARG and cancer endpoints provided low posterior probabilities for shared causal variants, it should be noted that this may also reflect limited power. The low posterior probabilities supporting either shared or distinct causal variants across several co-localisation analyses suggests that many of these analyses may have been too underpowered to support either of these configurations evaluated. Third, the low posterior probability of shared causal variants in ‘positive control’ co-localisation analyses for GLP1R and BMI could reflect distinct signalling mechanisms influencing type 2 diabetes and BMI in *GLP1R*, the presence of which would not necessarily influence the validity of this as an instrument for GLP1R signalling perturbation’s effect on glycaemic control [51]. Fourth, we were unable to evaluate the role of some glucose-lowering drug targets (i.e. DPP-4 and SGLT2) due to the absence of reliable genetic instruments for these targets. Fifth, our analyses were restricted to the examination of target-mediated (i.e. ‘on-target’) effects of glucose-lowering medications on cancer endpoints. Sixth, our analyses assume no gene–environment or gene–gene interactions and linear and time-dependent effects of drug targets on cancer risk. Seventh, though associations of genetically proxied PPARG perturbation and prostate and ER^+^ breast cancer risk attenuated towards the null in iterative leave-one-out analysis removing rs4135247 from the PPARG instrument, 95% CIs overlapped across models with and without this variant. Though this attenuation in association is consistent with sampling error, we cannot rule out the possibility that this attenuation was driven, in part, through horizontally pleiotropic mechanisms linking this variant to cancer risk. Eighth, though we found strong and suggestive evidence for associations of genetically proxied PPARG perturbation with ER^+^ breast cancer and prostate cancer risk, respectively, after applying a Bonferroni correction to account for multiple testing, we cannot rule out the possibility that these findings represent false-positive results. Ninth, the MR estimates reported represent long-term effects of target modulation in non-diabetic populations, whereas the clinical effects of these medications may be more pronounced among individuals with type 2 diabetes and could depend on length of medication use. Tenth, we cannot rule out the possibility that controls in cancer GWAS included individuals with latent, undiagnosed cancer, the presence of which would bias associations towards or away from the null, depending on the site of undiagnosed cancer and the relationship between drug targets examined and this cancer. We also cannot rule out the possibility of survival bias influencing genetic association estimates from cancer GWAS consortia that employed case–control study designs. If, for example, genetic variants used to instrument glucose-lowering drug target perturbation increased cancer risk and subsequent mortality prior to enrolment in a case–control study, this could induce an artificial ‘protective’ association between perturbation of this drug target and cancer risk. Finally, samples were restricted to individuals of European ancestry and therefore the generalisability of these findings to non-European populations is unclear.

In conclusion, we developed novel instruments for PPARG, ABCC8 and GLP1R using strict validation protocols and evaluated the association of genetically proxied perturbation of these targets with risk of cancer. In MR analysis we found weak evidence that genetically proxied PPARG perturbation was associated with a higher risk of prostate cancer and a lower risk of ER^+^ breast cancer. There was little evidence of co-localisation for these findings, a necessary precondition to infer causality between PPARG perturbation and these cancer endpoints, possibly reflecting either the absence of shared causal variants across type 2 diabetes liability and these cancer endpoints in *PPARG* or the low statistical power of these analyses. Further assessment of these drug targets using alternative molecular epidemiological approaches (e.g. using protein or expression quantitative trait loci or using direct circulating measures of these proteins) and/or studies using medical registry data (e.g. ‘target trial’ analyses) may help to further corroborate findings presented in this analysis. Finally, we found little evidence for an association of genetically proxied ABCC8 and GLP1R perturbation with risk of breast, colorectal, prostate or overall cancer risk.

## Supplementary Information

Below is the link to the electronic peer-reviewed but unedited supplementary material.

Supplementary file1 (PDF 226 KB)

## Data Availability

Summary genetic association data for select cancer endpoints were obtained from the public domain: breast cancer (https://bcac.ccge.medschl.cam.ac.uk/bcacdata/); and overall prostate cancer (http://practical.icr.ac.uk/blog/). Summary genetic association data for colorectal cancer can be accessed by contacting GECCO (kafdem at fredhutch.org). Summary genetic association data on advanced prostate cancer can be accessed by contacting PRACTICAL (practical at icr.ac.uk). Summary genetic association data on type 2 diabetes from Vujkovic et al [34] (can be accessed through dbGAP under accession number phs001672.v3.p1 (pha004945.1 refers to the European-specific summary statistics). UK Biobank data can be accessed by registering with UK Biobank and completing the registration form in the Access Management System (AMS) (https://www.ukbiobank.ac.uk/enable-your-research/apply-for-access).

## Abbreviations

ABCC8: Sulfonylurea receptor 1 (ATP binding cassette subfamily C member 8)
ALT: Alanine aminotransferase
AST: Aspartate aminotransferase
BCAC: Breast Cancer Association Consortium
CCFR: Colon Cancer Family Registry
CORECT: Colorectal Transdisciplinary Study
DPP-4: Dipeptidyl peptidase-4
ER: Oestrogen receptor
GECCO: Genetics and Epidemiology of Colorectal Cancer Consortium
GLP1R: Glucagon-like peptide-1 receptor
GWAS: Genome-wide association study
IRNT: Inverse rank normal transformed
MR: Mendelian randomisation
PPARG: Peroxisome proliferator activated nuclear receptor γ
PRACTICAL: Prostate Cancer Association Group to Investigate Cancer Associated Alterations in the Genome
SGLT2: Sodium–glucose cotransporter 2
